# Sex differences in glucocorticoid responses to shipping stress in Pekin ducks

**DOI:** 10.1016/j.psj.2021.101534

**Published:** 2021-10-13

**Authors:** V. Tetel, B. Van Wyk, G.S. Fraley

**Affiliations:** ⁎ Department of Animal Sciences, Purdue University, West Lafayette, IN, USA; † Department of Biology, Hope College, Holland, MI, USA

**Keywords:** sex difference, cortisol, corticosterone, stress, welfare

## Abstract

Some concerns have been raised recently about the assay of corticosterone vs. cortisol in poultry species. Thus, we tested the hypothesis that ducks secrete both glucocorticoids. First, we validated two commercially ELISA kits for the two glucocorticoids by first charcoal stripping duck serum in order to remove all steroid hormones. We ran serial dilutions of spiked, charcoal-stripped serum on kits of opposite glucocorticoid as well as a serial dilution using the respective ELISA buffer of the opposite assay kit. We found that the glucocorticoid standard curve in duck serum matched the respective curve in that kit's own buffer. However, when the opposite hormone was run in each kit in both duck serum or ELISA buffer, a near zero slope was obtained. Second, we further validated the presence of both glucocorticoids using mass spectrometry. Third, we tested the hypothesis that exogenous ACTH would stimulate the release of both corticosterone and cortisol. And, fourth, we tested the hypothesis that each glucocorticoid would have different serum levels in response to shipping stress. To test this hypothesis, we collected serum from 10 drakes and 10 hens from 2 flocks (N = 20 per time point per sex): 24 h prior to shipping, at shipping as ducks were walked off the truck, 24 h after shipping, and 1 wk after shipping. Data were analyzed by 2-way repeated measures ANOVA. Surprisingly, we also observed a sex difference in both glucocorticoid levels in that hens showed higher (*P* < 0.01) serum levels than did drakes at all-time points in response to either ACTH or transportation. Finally, no differences were observed in either glucocorticoid levels associated with shipping in either sex. The fact that both glucocorticoids are released in measurable amounts lends to the possibility that they may be differentially regulated, or at least there is a sex difference in the neural pathways associated with glucocorticoid release in ducks. Although corticosterone is the likely predominate glucocorticoid in ducks, serious attention should be given to the role of cortisol in poultry. Further consideration of sex, age, and timing of blood collection to stressor needs to be considered when assessing glucocorticoid levels in any avian species.

## INTRODUCTION

It has long been assumed that the avian hypothalamic-pituitary-adrenal axis (**HPA**) functions similarly to that of mammals. In mammals, the hypothalamic neurohormone, corticotropin releasing hormone (**CRH**) is produced by neurons within the paraventricular nucleus (**PVN**). In response to a stressor, it is released by synaptic boutons within the median eminence into the pituitary portal blood, where it binds to CRH receptors on corticotrophs to stimulate the release of adrenocorticotropic hormone (**ACTH**). Adrenal cortical cells thus release glucocorticoids, either corticosterone or cortisol, in a species-specific manner. However, there appears to be considerable differences in the avian HPA. The avian HPA uses a second neurohormone, arginine vasotocin (**AVT**), which is released from the median eminence to also stimulate pituitary corticotropes (Kuenzel et al., 2012). In birds, CRH is found primarily in a thalamic nucleus, the Nucleus of the hippocampal commissure (**NHpC**), while AVT is found primarily in the PVN—however, both neurohormones can be found in both diencephalic nuclei (Chen et al., 2000). Finally, it has generally been believed that the adrenal cortex primarily secretes corticosterone in all birds, including the duck (Holmes et al., 1972; Harvey et al., 1980). However, some research has suggested that birds may also secrete both, or either, glucocorticoid in response to ACTH (Ohkura et al., 2000; Schmidt and Soma, 2008; Schmidt et al., 2009, 2010; Caulfield and Padula, 2020).

Both primary glucocorticoids are synthesized from a common pathway that begins with cholesterol and pregnenolone. However, after synthesis to pregnenolone, the synthetic pathways diverge to produce either corticosterone or cortisol. Although there may be some cross-over between the biosynthetic pathways, the synthesis of one of the glucocorticoids does not depend upon the synthesis of the other. In general, glucocorticoids do have similar functions regardless of the species. As the name suggests, a primary goal is to increase blood glucose levels in response to a stressor, in order to prepare an animal for fight or flight (Thompson and Lippman, 1974). In migratory birds, glucocorticoids are associated with nocturnal restlessness, increased feeding behaviors, and adipogenesis related to the migratory drive (Eikenaar et al., 2018; Tsvey et al., 2019). These factors may be important for our poultry species with migratory ancestors, such as the duck. Nonstressful events also increase glucocorticoid release in numerous species such as a mild increase in physical activity (Campbell et al., 2015). There is quite a bit of variability in terms of the predominate glucocorticoid (**GC**) in animals. For example, many mammals and fish utilize cortisol as well as some rodents (guinea pigs, squirrels), however other rodents, such as rats and mice, utilize corticosterone. However, research has concluded that some species do in fact utilize both glucocorticoids as a part of physiological homeostasis, including bottlenose dolphins (Ortiz and Worthy, 2000). Birds are assumed to also utilize corticosterone. However, some evidence does exist that suggests that birds may also utilize cortisol (Schmidt and Soma, 2008; Caulfield and Padula, 2020).

We tested the hypothesis that ducks utilize both glucocorticoids. The purpose of these experiments was first to validate ELISA assays for each duck GC, second to determine GC response to ACTH stimulation, and third to determine GC release around transportation of ducks. Our results suggest that although corticosterone is likely the primary GC in ducks, there may be a physiological role for cortisol as well.

## MATERIALS AND METHODS

### Experiment 1: Validation of ELISAs for Corticosterone and Cortisol

We wished to use ELISAs for experimental procedures due to logistics and large number of samples collected from these and future studies. ELISA kits for corticosterone and cortisol were obtained from Cayman Chemical (Ann Arbor, MI). In order to validate the kits, we first charcoal-stripped 20 mL of pooled duck serum in order to remove all steroids from the sample. Second, we produced 3 standard curves for each kit. The first curve was produced using the manufacturer's ELISA buffer and respective control steroid. The second standard curve was produced using the steroid for each respective kit substituting the charcoal stripped serum in place of the ELISA buffer. The third standard curve was produced again with charcoal-stripped serum but with the opposite steroid—cortisol used with the corticosterone kit and vice versa. All standard curve ranges were produced following the manufacturer's recommendation. In addition, we also ran the charcoal stripped serum in order to confirm the removal of GCs. All remaining steps of the ELISA were completed following the manufacturer's instructions and plates read at 405 nm using SynergyLx (Biotek, Winooski, VT).

### Experiment 2: Targeted Analyses of Cortisol and Corticosterone in Plasma Extracts Using Agilent Triple Quadruple Mass Spectrometry (QQQ) for Verification of ELISA

In order to confirm ELISA validation, we ran additional samples (N = 20 per sex) using mass spec to validate presence of both glucocorticoids in each sample. Samples were stored at −80°C prior to extraction and analysis and 10% of total samples assayed per treatment group by the Bindley BioScience Center at Purdue University. At the time of analysis, each plasma sample was thawed, and 0.2 mL transferred to an extraction tube. To each sample 10 µL of an internal standard mixture containing 5 ng of deuterated corticosterone (d_8_-corticosterone solution in methanol) and 0.05 ng of deuterated cortisol (d_4_-cortisol solution in methanol) was added to the plasma and vortexed for 1 min. Next 1 mL of ethyl acetate was added to extract corticosterone and cortisol from the aqueous plasma sample. The samples were vortexed for 10 min and centrifuged at 13,000 g for 10 min. The top organic layer was collected and transferred to a new tube for drying. The samples were dried in a rotatory evaporation device at 45°C for 3 h. Each sample was then derivatized with 50 µL of Amplifex keto reagent (# 4465962, AB Sciex, Framingham, MA) according to the kit directions just prior to instrument analysis. The internal standards d_8_-corticosterone (# C695702) and d_4_-cortisol (# C696302) were purchased from Toronto Research Chemicals (Ontario, Canada).

An Agilent 1260 Rapid Resolution liquid chromatography (**LC**) system coupled to an Agilent 6470 series QQQ mass spectrometer (MS/MS) was used to analyze corticosterone and cortisol in each plasma sample (Agilent Technologies, Santa Clara, CA). An Agilent Eclipse plus C18 2.1 mm x 50 mm, 1.8 µm column was used for LC separation. The buffers were (A) water + 0.1% formic acid and (B) acetonitrile + 0.1% formic acid. The linear LC gradient was as follows: time 0 min, 10% B; time 1.0 min, 10% B; time 1.5 min, 25% B; time 21.5 min, 35% B; time 22 min, 100% B; time 23 min, 100% B; time 24 min, 10% B; time 30 min, 10% B. The flow rate was 0.3 mL/min. Corticosterone eluted at 6.6 min and cortisol at 5.8 min. Multiple reaction monitoring was used for MS analysis. The data were acquired in positive electrospray ionization (**ESI**) mode according to Table 1. The jet stream ESI interface had a gas temperature of 325°C, gas flow rate of 8 L/min, nebulizer pressure of 45 psi, sheath gas temperature of 250°C, sheath gas flow rate of 7 L/min, capillary voltage of 4,000 V in positive mode, and nozzle voltage of 1,000 V. The ΔEMV voltage was 500 V. Agilent Masshunter Quantitative analysis software was used for data analysis (version 8.0). For quantitation of corticosterone/d_8_-corticosterone, the transition 461.3→402.2/469.3→410.2 was used. For cortisol/d_4_-cortisol, the transition 477.3→418.3/481.3→422.3 was used. Table 1 shows the reaction monitoring table for data acquisition.

**Table 1:** Multiple reaction monitoring table for data acquisition.

Compound name	Precursor ion (m/z)	Product ion (m/z)	Collision energy (V)
Corticosterone	461.3	402.2	15
d8-Corticosterone	469.3	410.2	15
Cortisol	477.3	418.3	15
Cortisol	477.3	388.2	35
d4-cortisol	481.3	422.3	15
d4-cortisol	481.3	392.3	30

### Experiment 3: Effects of ACTH Stimulation on Glucocorticoid Release in Drakes and Hens

Young adult (16 wk; ∼4 kg) male and female ducks were obtained from Maple Leaf Farms, Inc and housed at Purdue's Animal Sciences Research and Education Center (**ASREC**). They were given access to feed for 8 h per day and ad lib access to water following standard procedures for this age duck. In order to determine if ACTH could stimulate both GC release from the duck, we injected a standard veterinary dose of artificial ACTH (Cosyntropin; ACTH1-24; 0.0625 mg/in 1.0 mL saline), or saline as control, intramuscularly (N = 10/sex/treatment). This dose has been commonly used in many species of animals during ACTH challenge tests including humans (McGregor et al., 2002; Hamilton and Cotton, 2010), ducks (Nilsson et al., 2008), and chickens (Siegel and Beane, 1961). Blood was collected from the tibial vein at time 0-, 1-, and 2-h surrounding ACTH injection. Serum was collected and stored at -20°C until analyzed by ELISA. ELISA was the chosen protocol for hormone assessment due to the long time (40–50 min) it takes to run each sample one at a time on mass spectrometry. The long duration and repeated assays over many weeks would have inevitably increased intersample variability.

### Experiment 4: Effects of Transportation Stress on Serum Glucocorticoids

The transportation experiment was done onsite at Maple Leaf Farms from two separate commercial barns. We assessed developer (14 wk of age) drakes and hens 24 h prior to transportation to the breeder barn (pretransport), as they walked off the truck at the breeder barn (Transport), and 24 h (Transport +24) and 1 wk (Transport + 1 wk) after transportation (N = 10 per sex/time point/barn; final N = 20 per sex/time point). Transportation was from the developer barn to the breeder barn and took approximately 1 h. The first ducks off the truck at the destination were collected for blood collection. For all steroid assays, blood was collected from the tibial vein into serum separator tubes, centrifuged and serum stored at -20°C until assayed. All experiments were approved by the Purdue University Animal Care and Use Committee (PACUC).

### Statistical Analyses

Data were analyzed using MacJMP Pro 15 (SAS). In experiment #1, linear regressions were produced and analyzed using MacJMP. The duck was considered the statistical unit for the remaining experiments. In experiment #2 and #3, data for each GC were analyzed using a 3-way repeated measures ANOVA (treatment x sex x time). We did not statistically compare between hormones as they are measured using slightly different methods and at different times, thus a direct comparison would not be appropriate. In experiment #4, we could not utilize the same ducks for each time point due to the large number of ducks present in the commercial barn, thus we utilized a 2-way ANOVA (sex x time) without repeated measures. Post hoc analyses were done by a Fisher's PLSD and a *P* < 0.05 considered significant. All data are presented as means +/− standard errors.

## RESULTS

### Experiment 1: Validation of ELISAs for Corticosterone and Cortisol

The standard curve for the corticosterone kit using charcoal-stripped duck serum showed similar results as the standard kit using the provided ELISA buffer (R^2^ = 0.9770 and 0.9879, respectively). Running the cortisol standard curve in duck serum using the corticosterone kit resulted in negligible results (R^2^ = 0.0443). Similarly, the standard curve for the cortisol kit using charcoal-stripped duck serum showed similar results as the standard kit using the provided ELISA buffer (R^2^ = 0.9943 and 0.9775, respectively). Running the corticosterone standard curve in duck serum using the cortisol kit resulted in no results (y = 0, R^2^ = N/A). The charcoal stripped samples in both kits showed no measurable levels of each glucocorticoid, respectively. Sample standard curves are illustrated in Supplemental Figure 1.

### Experiment 2: Targeted Analyses of Cortisol and Corticosterone in Plasma Extracts Using Agilent Triple Quadruple Mass Spectrometry (QQQ) for Verification of ELISA

**Figure 1:**
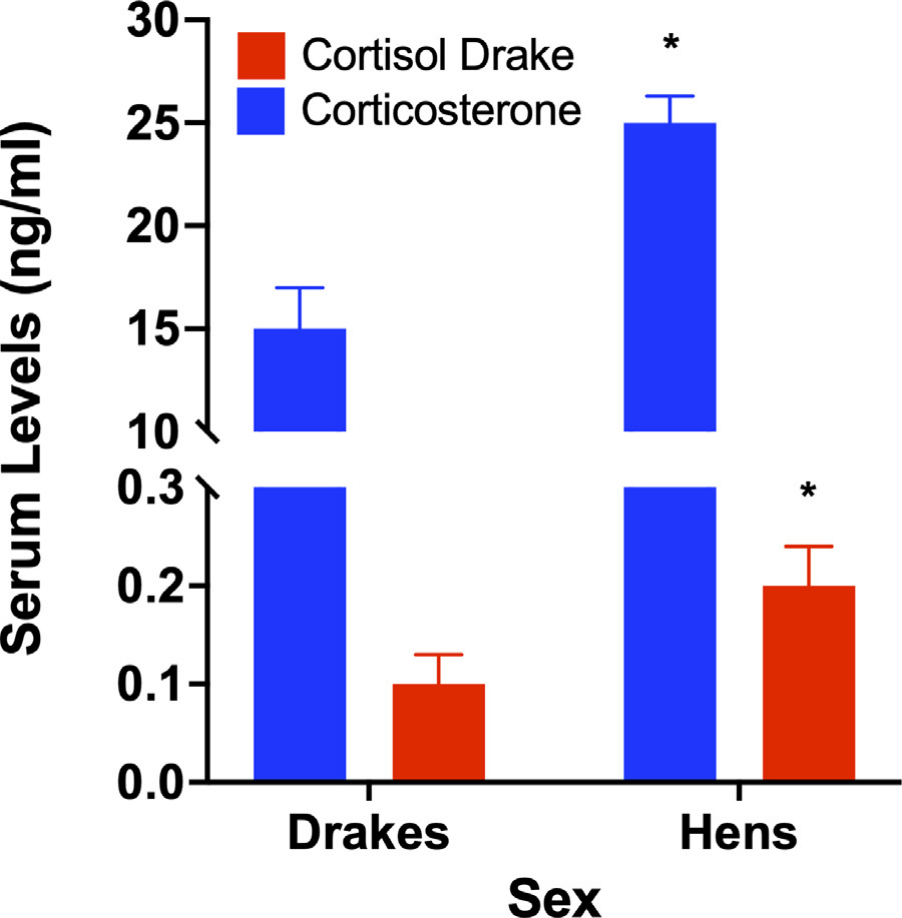
Glucocorticoid assay in duck serum. Using Triple Quadruple Mass Spec we observed both glucocorticoids in duck serum and a significant sex difference in levels of each glucocorticoid where hens showed greater serum levels then do drakes.* = *P* < 0.05.

Samples analyzed for corticosterone and cortisol using mass spectrometry resulted in similar patterns of change, albeit greater concentrations than noted in the ELISA. The concentration differences are likely due to the fact that mass spectrometry has a much greater sensitivity then ELISA. However, the differences at different concentration remain linear between the two techniques (data not shown). Further, the mass spectrometry data still show a significant sex difference (*P* < 0.05) for each glucocorticoid as observed in the ELISAs (Figure 1).

### Experiment 3: ACTH Stimulation Test

**Figure 2:**
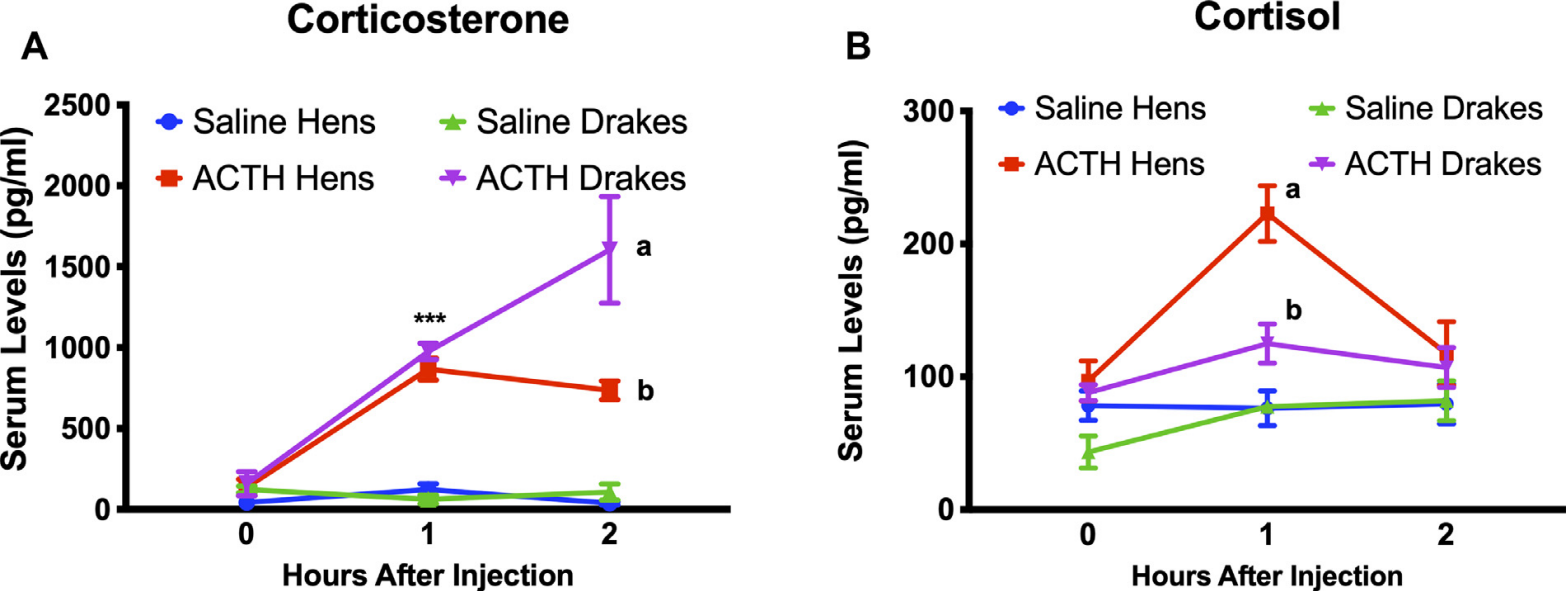
ACTH stimulation of glucocorticoids in duck. ACTH caused a significant increase in (A) corticosterone and (B) cortisol compared to saline control, albeit in different patterns of release. Further, a significant sex difference was observed with hens showing increased cortisol, but not corticosterone, compared to drakes, although drakes did show an increase at 2 h. *** = sex difference in GC levels at *P* < 0.001, letters indicate statistically different groups at *P* < 0.05.

ACTH intramuscularly (**IM**) resulted in significant interaction among independent variables (*P* < 0.01). We observed a significant increase in serum corticosterone in both sexes at 1 h compared to saline IM (*P* < 0.05). Drakes continued to rise at 2 h to be significantly (*P* < 0.01) different from both ACTH injected hens and controls. ACTH IM resulted in significant increase in serum cortisol levels 1 h after injection in both sexes, however hens showed significantly greater levels of cortisol compared to drakes. By two h postinjection, both hens and drakes showed similar levels of serum cortisol compared to saline injected animals. Figure 2 illustrates these results.

### Experiment 4: Shipping Stress

**Figure 3:**
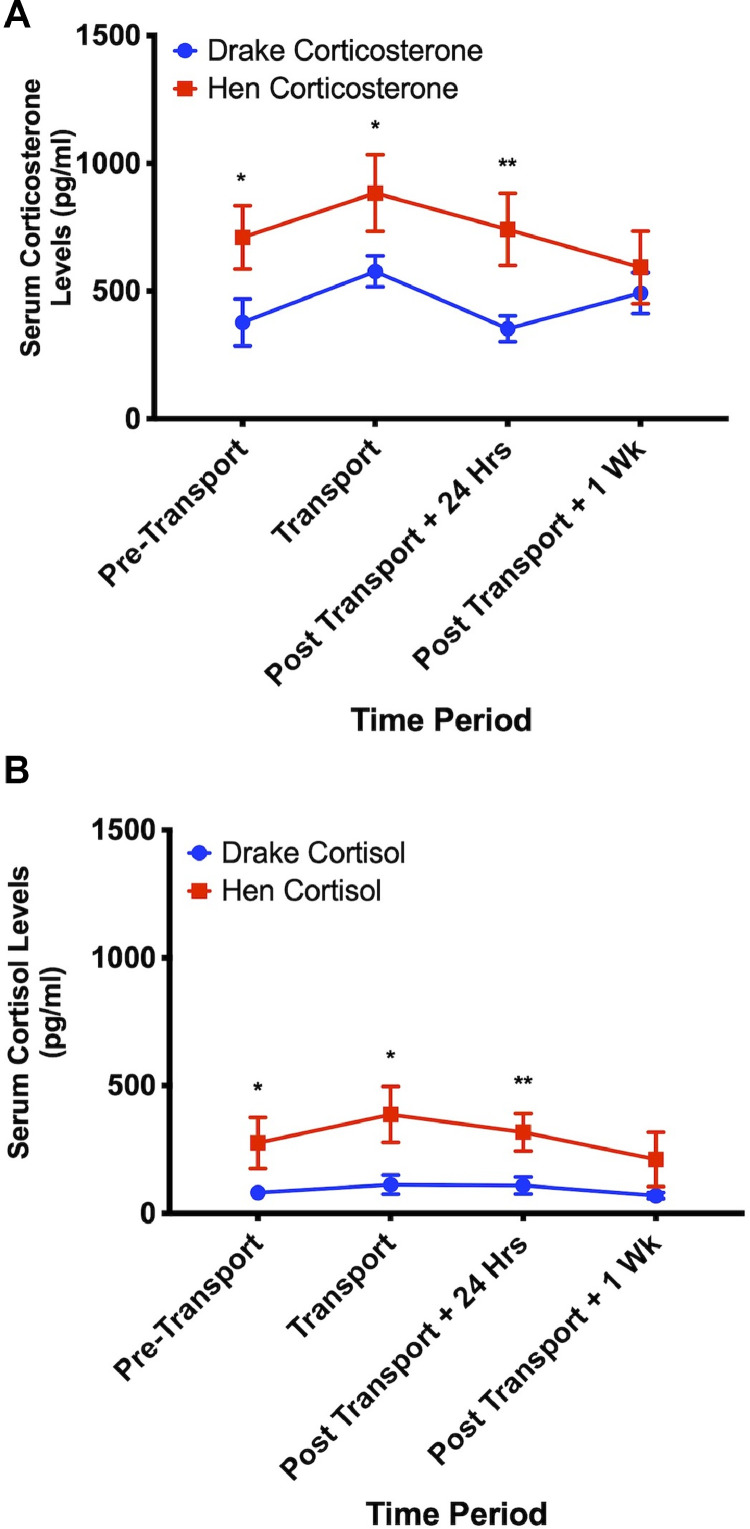
Glucocorticoid response to transportation. Although no significant differences were observed in either (A) corticosterone or (B) cortisol secretion at transport compared to pre- or post-transport time points, a significant sex difference was observed in both glucocorticoids with hens showing greater serum levels then drakes. * = *P* < 0.05, ** = *P* < 0.01.

Although the two glucocorticoids were not analyzed against each other due to their being processed in separate ELISA kits, cortisol typically was released at about 1/3 of the level of corticosterone in both sexes. There was a significant interaction between sex and time (*P* < 0.01) for both hormones. Hens showed significantly (*P* < 0.01) higher levels of serum corticosterone compared to drakes at all time points except 1 wk following transport. At each time point, hens also showed greater (*P* < 0.01) levels of serum cortisol compared to drakes. There was a slight but nonsignificant increase in both serum corticosterone and cortisol associated with transportation in hens and drakes. Figure 3 illustrates these results.

## DISCUSSION

The purpose of this study was to investigate if both glucocorticoids, corticosterone and cortisol, are released in response to ACTH stimulation or to an external stressor, shipping stress. In order to accomplish this goal, we first verified the use of an ELISA for each GC through charcoal stripping and readdition of standard curves. Further, we also validated results through mass spectrometry. We found our specific ELISA was a reliable tool to measure each respective GC with negligible cross-reactivity. Further, we found that both corticosterone and cortisol are secreted in measurable levels, albeit cortisol at only about 1/3 of the serum levels compared to corticosterone. Interestingly, we observed a significant sex difference in circulating levels of corticosterone and cortisol at nearly every time point assessed, with hens showing greater levels than observed in drakes.

Historically, ACTH stimulation has been utilized in numerous species of birds to elicit adrenal responses. Hall and Koritz (1966) demonstrated that ACTH elicited a conversion of cholesterol to corticosterone in chicken adrenals, noting the similarity to mammals, and further supported by Frankel et al. (1967) in cockerels. Others (Siegel and Siegel, 1966; Siegel, 1968) showed differences in responsiveness to ACTH on corticosterone release and other physiological factors influenced by strain of chicken as well as age. Similar observations have been made in the pigeon, egret, and myna (Bhattacharyya et al., 1967). Siegel reported similar changes in blood cell counts in chickens treated with ACTH or cortisol (Siegel, 1968). Bradley and Holmes (1972) addressed the importance of the adenohypophysis and ACTH in regulation of the adrenal glands in the duck. All of these observations are related to the Selye hypothesis in that there are 3 stages to the physiological response to stress: first, the alarm or neurogenic response that involves the sympathetic nervous system; then second, the adaptive, or humoral, phase in which the adrenal cortex secretes glucocorticoids in an attempt to adapt to the damage or stressor; and third, if animals do not adapt, then they enter exhaustion, deterioration and ultimately death (Selye, 1937, 1950, 1975). Thus, the adaptation phase that involves glucocorticoids occurs over time beyond the onset of the stressor, as is observed in our study which found that the glucocorticoid response to ACTH occurred 1 to 2 h after injection. Further, the levels of glucocorticoids secreted in response to ACTH in our study were comparable to those reported previously by others (Gross, 1990) and reported previously by our lab (Campbell et al., 2015, Campbell et al., 2015; Haas et al., 2017). However, the glucocorticoid response to transportation stress in our ducks was less clear, as has also been suggested by others.

In chickens, transportation can have multiple effects on circulating corticosterone levels. Some studies have shown that transportation has no effects on circulating corticosterone (Kannan et al., 1997; Zhang et al., 2009). Other studies have shown that transportation increases circulating corticosterone levels (Al-Aqil et al., 2013) while yet others have shown that corticosterone levels are reduced as a result of transportation (Vosmerova et al., 2010). In our study there was a slight but nonsignificant increase in serum corticosterone levels associated with transport, but we did see a very clear sex difference with hens having higher levels of corticosterone then males at nearly every time point. The sex differences were not replicated for corticosterone following ACTH stimulation, but were with serum cortisol levels. The lack of corticosterone effect was likely due to the high dose of ACTH. Future studies will employ a dose response curve to better determine the effects of ACTH on corticosterone and cortisol release in the duck. The lack of significance in our study associated with transportation as well as conflicting reports in other studies may be due to the timing of the blood collection relative to the onset of the stressor. Numerous studies have also shown that females tend to have higher levels of glucocorticoids in mammals than do males (Kitay, 1963; Gaskin and Kitay, 1970; Kant et al., 1983; Haleem et al., 1988; Heinsbroek et al., 1990; Yoshimura et al., 2003; reviewed by (Kudielka and Kirschbaum, 2005)). Small and Schoech (2015) demonstrated a sex difference in glucocorticoid programming for adult responses in Florida scrub jays. Similar observations have been made in Great Tits (Van Der Meer and Van Oers, 2015). Other stressors, such as metabolic/nutritional stressors, shackling, and stocking density, among others, have also resulted in conflicting observations on the effects of circulating corticosterone (reviewed by Scanes (2016)). It is not clear in these numerous studies if there were consistencies in the timing of the blood draws following the stressor, blood sampling time relative to oviposition (Soliman and Huston, 1974), the circadian rhythmicity of HPA function (Majsa et al., 1976), or in taking into consideration the sex or age of the birds. It is also possible that the differences in experimental design, crating vs. not crating, mock transportation vs. actual, or different methods in handling the birds. Regardless, it appears that the most profound sex differences in glucocorticoid function are due to gonadal steroid-glucocorticoid receptor interactions in mammals (reviewed by Kudielka and Kirschbaum (2005)). In our ACTH study, both sexes showed similar baseline GC levels, however, in the shipping stress experiment hens showed significantly higher baseline GC compared to drakes. During the shipping stress study, we collected blood from ducks that were the last to be loaded on the truck so that we could capture the first to come off and more accurately assess the time of shipment. It may be possible that due to vocalizations made by ducks being loaded on the truck caused the last to be loaded hens to begin showing signs of stress, although the relationship between vocalizations and physiology has never been assessed in any poultry species. However, this has yet to be systematically studied in poultry. Interestingly, our study also showed cortisol being released in response to both transportation and to ACTH stimulation, also in a sex-dependent manner.

The idea that cortisol may play a role in the stress response in birds is not novel. Kalliecharan (1981) demonstrated that ACTH did not selectively act on the corticosterone or cortisol synthetic pathway in immature chicks, and rather increased both glucocorticoids. Observations in the cockatoo also revealed sex differences in response to exogenous ACTH treatment. However, that study did not observe a cortisol response to exogenous ACTH (Walsh et al., 1985), similar to observations in other psittacines (Lothrop et al., 1985). In contrast, Zenoble et al. (1985) did show that exogenous ACTH stimulated cortisol in parrots. Thus, the role of cortisol may be species dependent, or our ability to measure cortisol actions may be species dependent. A recent study showed that cortisol could be a reliable indicator of an acute stressor in mule ducks (Flament et al., 2012). They showed that 45 min after ACTH treatment or force-feeding, corticosterone levels did not change, but cortisol levels did increase (Flament et al., 2012). Another study showed that there are intracellular and membrane-bound, cortisol-specific receptors in developing zebra finches. This study also showed that cortisol appears to be the primary glucocorticoid to bind to bursal tissue, and binds with high affinity to a neural membrane receptor (Schmidt et al., 2010). The same lab showed that restraint stress in zebra finches had minimal effects on levels of both GCs on the day of hatch, but significantly increased both GCs at d 10, again showing age related differences in GC function (Schmidt et al., 2009). Further studies have suggested that cortisol may be produced within other organs, such as spleen and bursa of birds, in order to directly affect immune-cell development or function (Taves et al., 2017). Although beyond the scope of our current study, future studies in the duck will also include analyses of immune function and lymphoid tissues, and the possibility of de novo GC synthesis as described above in the zebra finch. A recent study has demonstrated that cortisol, not corticosterone, is present in eggs and may be the preferred GC to assess hen welfare or stress (Caulfield and Padula, 2020).

In summary, we have thoroughly verified ELISA kits to measure corticosterone and cortisol in duck serum. We have demonstrated a sex difference in both GCs in which hens have greater circulating levels than do males, particularly in response to exogenously administered ACTH. Finally, we have demonstrated a nonsignificant increase in both GCs in response to transportation; the lack of significant increase may have been due to the timing of blood collection relative to the onset of the putative stress. Our data suggest that although corticosterone may be the predominate GC in ducks, cortisol is also responsive to stimuli and should be more thoroughly investigated as a tool to measure welfare of our poultry species.

## Appendix Supplementary materials

Supplemental Figure 1. Sample standard curves during ELISA validation. Validation of the corticosterone ELISA using (A) corticosterone standard curve in ELISA buffer, (B) cortisol standard curve in ELISA buffer, and (C) corticosterone standard curve in charcoal-stripped duck serum. Validation of the cortisol ELISA using (D) cortisol standard curve in ELISA buffer, (B) corticosterone standard curve in ELISA buffer, and (C) cortisol standard curve in charcoal-stripped duck serum. Image, image 1

## Footnotes

Supplementary material associated with this article can be found in the online version at doi:10.1016/j.psj.2021.101534.
