# Supplementary figures and images for: Sex differences in glucocorticoid responses to shipping stress in Pekin ducks
Source: Poult Sci. 2021 Oct 13;101(1):101534. doi: 10.1016/j.psj.2021.101534 (PMC8626681; doi:10.1016/j.psj.2021.101534)

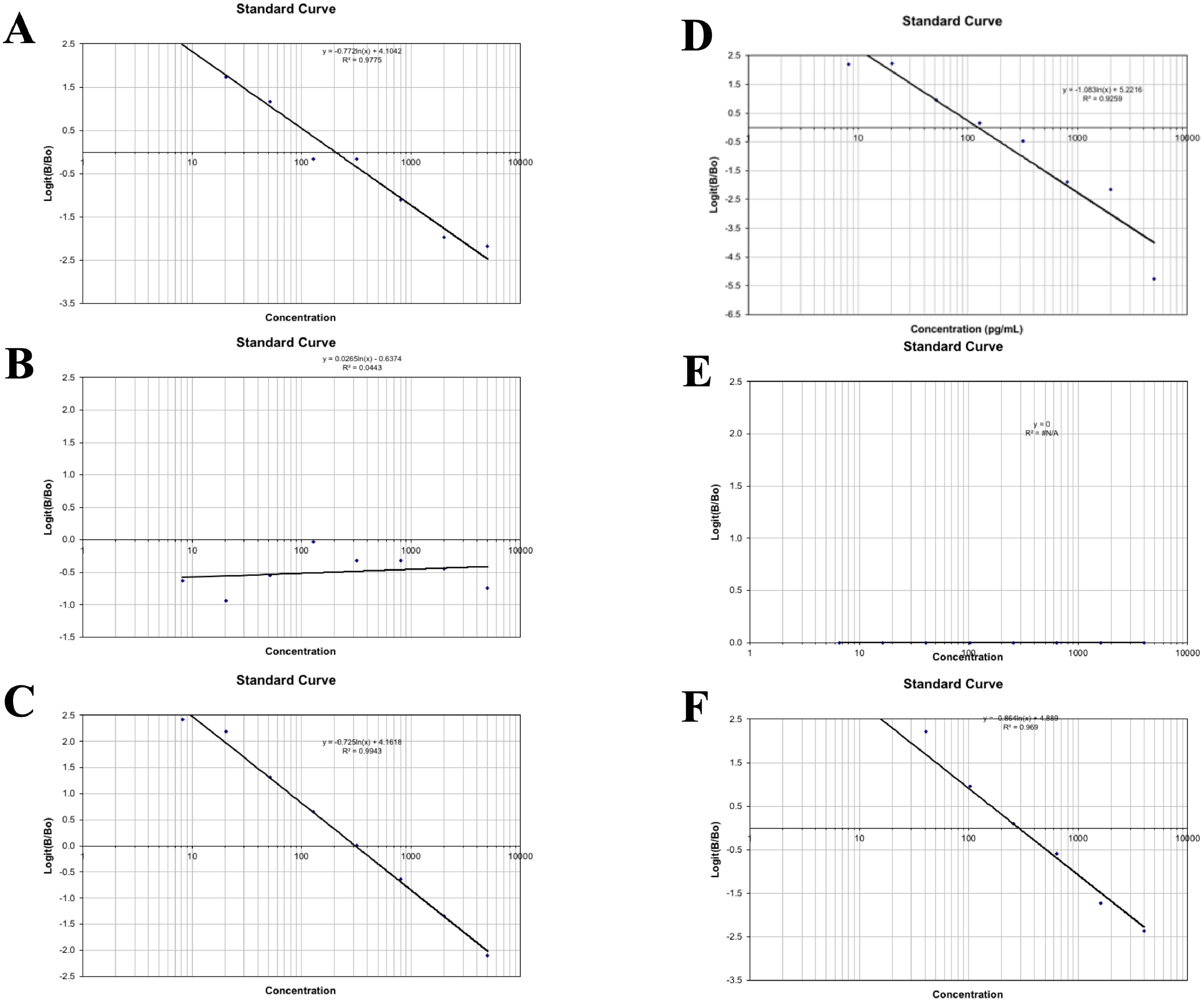

Supplement: Supplementary file 1 — Supplemental Figure 1. Sample standard curves during ELISA validation. Validation of the corticosterone ELISA using (A) corticosterone standard curve in ELISA buffer, (B) cortisol standard curve in ELISA buffer, and (C) corticosterone standard curve in charcoal-stripped duck serum. Validation of the cortisol ELISA using (D) cortisol standard curve in ELISA buffer, (B) corticosterone standard curve in ELISA buffer, and (C) cortisol standard curve in charcoal-stripped duck serum. [file mmc1.jpg]
